# The Powder Diffraction File: Past, Present, and Future

**DOI:** 10.6028/jres.101.027

**Published:** 1996

**Authors:** Deane K. Smith, Ron Jenkins

**Affiliations:** JCPDS-International Centre for Diffraction Data, Newtown Square, PA 19073

**Keywords:** CD-ROM, ICDD, PDF, phase identification, powder data, x-ray diffraction

## Abstract

The Powder Diffraction file has been the primary reference for Powder Diffraction Data for more than half a century. The file is a collection of about 65 000 reduced powder patterns stored as sets of *d*/*I* data along with the appropriate crystallographic, physical and experimental information. This paper reviews the development and growth of the PDF and discusses the role of the ICDD in the maintenance and dissemination of the file.

## 1. Introduction

The Powder Diffraction File (PDF) has been the primary reference for powder diffraction data since the Dow Chemical Company allowed its data, first published by Hanawalt, Rinn, and Frevel (1938) [1], to be reprinted in 3″ × 5″ card format under the auspices of the American Society for Testing and Materials (ASTM). The importance of the diffraction information was recognized by the early pioneers in the field who formed a Joint Committee for Chemical Analysis by Powder Diffraction Methods co-sponsored by ASTM Committee E-4, the Crystallographic Society of America, and the British Institute of Physics. This reprint appeared in 1941 as Set 1 of the PDF. Since this date, a total of 45 sets have been published with over 71 000 diffraction patterns.

The primary information in the PDF is the collection of *d-I* data, the *d*-spacing (d) determined from the angle of diffraction, and the intensity (*I*) experimentally measured under the best possible conditions for a phase-pure material. These data provide a “fingerprint” of the compound because the *d*-spacings are fixed by the geometry of the crystal and the intensities are dependent on the elements and their arrangement in the crystal structure. Thus, the *d-I* data may be used for identification of unknown materials by locating matching *d-I* data in the PDF with the *d-I*’s obtained from the unknown. Identification is the most common use for the PDF, but the presence of considerable supporting information on each compound allows further characterization of the compound under study. Examination of the crystal data, Miller indices, intensity values and scale factors, physical property data, and the reference provide useful information to further the knowledge of the sample. A detailed description of the PDF may be found in Jenkins and Smith (1985 and 1987) [2].

To meet the needs of diffractionists, the PDF is continually revised and updated with new and improved information. The increased use of computers for analysis of diffraction data and the increasing size of the file require greater accuracy of the reference information. Both improved instrumentation and more sophisticated analyses have placed more stringent requirements on how data are collected, reviewed and presented, particularly in computer-readable form. This paper will review the history and present status of the PDF and examine the current activities of the Joint Committee on Powder Diffraction Standards (JCPDS)-International Centre for Diffraction Data (ICDD) with respect to future improvements in the PDF and related products.

## 2. The History of the PDF

**Fig. 1:**
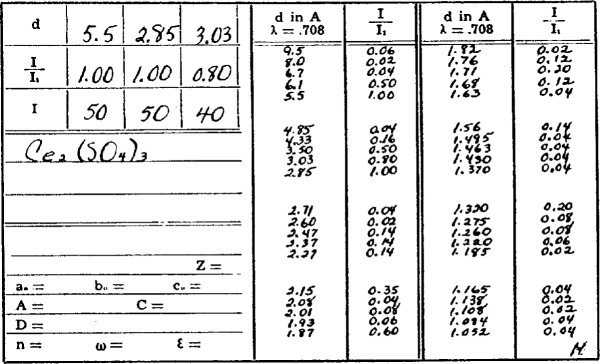
Card image for Ce_2_(SO_4_)_3_ from PDF Set 1 as issued in 1941.

The original form of publication of the PDF was as 3″ × 5″ file cards formatted so that the index lines, the three strongest and the *d-I* pairs for the three largest *d*-spacings, were placed at the top edge of the card and the full *d-I* list and supporting information filled the remaining space, Fig. 1. This format was devised to use the search scheme proposed by Hanawalt and Rinn (1936) [3]. By placing the cards in a drawer in “Hanawalt” order, the user could thumb through the cards examining the index lines to find a match. As the number of cards increased with Sets 2 (1945) and 3 (1949), the stack of cards became quite large. The larger number of entries made the PDF more useful because of the increased coverage, but it was also cumbersome to use for identification. A book-form Search Manual was devised in 1950 by the PDF editor, W. P. Davey. This manual was prepared by typing one-line search entries with the three strongest lines, the chemical formula and the PDF number for each compound, ordering the strips in a Termitrex holder, and photographing the assembly for offset printing. With the publication of Set 3 in 1949, the PDF contained about 2500 entries. Set 4 (1953) and Set 5 (1955) brought the PDF up to 4500 entries and marked the end of the first era of PDF history.

In the time period 1941–1955, the PDF overcame many of the problems of a fledgling database. The financial support was initially based on contributions from industry and scientific societies, but by 1955 the PDF was becoming self-supporting through sales revenue. The offices of the Joint Committee moved from the Pennsylvania State University to a location in down-town State College, PA, USA for 3 years, and then to ASTM headquarters in Philadelphia, PA, USA. An Associateship was established at the National Bureau of Standards (U.S.) to collect data under controlled and reproducible conditions. This Associateship provided *d-I* data on common, important compounds to replace multiple entries in the earlier sets and greatly improved the accuracy of the PDF. These data were collected on diffractometers rather than cameras. Some of the individuals who made significant contributions at this early stage were: W. P. Davey, A. S. Beward, W. L. Fink, H. W. Pickett, A. J. C. Wilson, and L. L. Wyman. The next 10 years saw regular issues of new sets of d-I data with 1000 to 1500 new entries each year. Several new search aids were developed including Keysort and IBM cards, the Fink Index, the Matthews Index, and the Fink Graphical Index. Of these hard copy products, only the Fink Index has survived the test of time, and it has not been prepared since 1982 except for some subfiles of the PDF. In 1962, the *d*-*I*’s, formulas, and PDF numbers were first keyboarded for a computer-readable database. The presence of these data in computer-readable form allowed the 1965 Search Manual to be computer composed. The availability of the data on magnetic media spurred the development of computer-based search/match schemes and the beginning of the next era. Three important individuals during this era were the editors of the PDF, G. W. Brindley and J. V. Smith, and Computer Consultant, G. G. Johnson, Jr.

During the first two stages, the Joint Committee evolved from a small group of volunteers who initiated the PDF to a small paid staff operating at ASTM and acting as editors under the guidance of a committee of volunteer scientists who reviewed the progress of this staff and recommended policies and products. By 1965, there were three committees: Finance, Technical, and Editorial. The membership of the Joint Committee was about 30, and the meetings were simple, with each subcommittee meeting on a separate day during a week each summer. Most members were active on all committees A Board of Directors consisting of four members directed the operations: W. L. Fink, Chairman; R. G. Simard, General Manager; A. W. Danko, Chairman, Technical Committee; and L. L. Wyman, Treasurer.

The year 1969 was a major turning point in the history of the PDF. For a variety of reasons, the operations were moved from ASTM headquarters to another location in Philadelphia, and the Joint Committee was incorporated as a separate corporation in the State of Pennsylvania. The name was changed to the Joint Committee on Powder Diffraction Standards (JCPDS). In 1971, the JCPDS moved to its headquarters in Swarthmore, PA where it remained for 20 years. The size of the staff increased as production increased, and the number of volunteers also increased. The number of meetings increased to two each year, and the additional subcommittees required more time and concurrent sessions. Currently, there are many specific Subcommittees of the Technical Committee alone. Grants-in-Aid was established to sponsor the collection of new data at universities, and these projects have become major sources of new data.

The period 1965–1978 saw several new products. The JCPDS sponsored an on-line search service, 2dTS, as the first computer-based search/match activity using the computer-readable database. Several individuals also developed programs for identifying crystalline phases using the *d-I* computerized information. Part of this development was spurred by the prospect of soft-landing a diffractometer on the moon. Automated powder diffractometers (APDs) were developed by many manufacturers using the PDF for phase identification as part of the supplied software. The *d-I* database provided the basis for the search/match procedures, but the final evaluation of the results required consulting hard copy forms of the PDF for the supporting information. Computer storage limitations were the main reason why the information in the database was usually distributed and utilized on magnetic tape. In spite of increased information density on the magnetic tape, the PDF soon exceeded the capacity of a single tape. Other new products included several hardcopy Subfiles, such as Minerals, and Metals and Alloys, to make the PDF more amenable to identification in these specialty areas.

In 1978, the corporation name was changed to JCPDS-International Centre for Diffraction Data (ICDD) to reflect the international use of the PDF and the increased diversity of products with the marketing of NIST Crystal Data. The role of the computer in future products was recognized, and a project was begun to keyboard and re-evaluate all the data which comprise the PDF, including the supporting documentation. This re-evaluation checked for internal consistency of unit cells, *d*-spacings, and symmetry wherever possible. This project was completed in 1984, and the resulting database allows a computer display of the data-card image, so no hard copy form is needed. The increased packing density of information on computer media has permitted the distribution of this database in several forms including the CD-ROM. The increased use of the computer-based PDF and the problems of storage of the classic cards prompted the JCPDS-ICDD to discontinue the production of cards in 1987, leaving books as the only hardcopy form published.

**Fig. 2:**
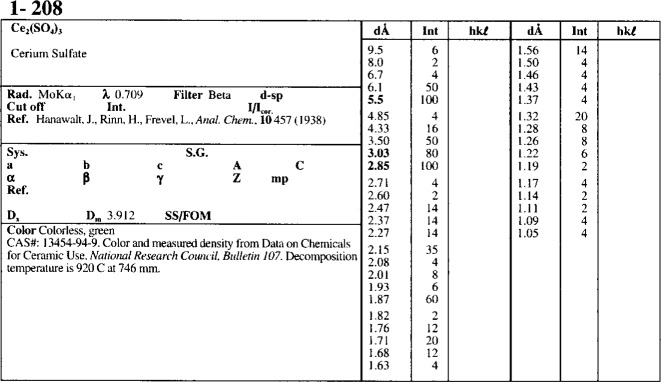
Card image for Ce_2_(SO_4_)_3_ from PDF Set 1 as printed in the 1995 format.

As of October 1995, the PDF contains 45 data sets with 71 067 diffraction patterns. These patterns are divided into the categories presented in Table 1. Many patterns are classified in more than one subfile. Each individual data set contains, as a minimum, a list of the *d-I* pairs, the chemical formula, the mineral name if appropriate, a unique identification (PDF) number, and a reference to the original source of the data. In addition to this information, supplemental data may include: the crystal unit cell and symmetry, Miller indices, physical constants, experimental details, and other comments such as preparation techniques and the chemical analysis. Figure 2 shows a modern card image which is to be compared with Fig. 1 to illustrate the changes that time and changing needs have implemented. In addition to a change in the format, most early data have been replaced with more accurate and expanded data and more documentation, including the crystal parameters where they were not known before.

In accordance with the nonprofit classification of the Corporation, the research and development activities include sponsoring programs for the improvement of the quality of diffraction data. In addition to the Associateship established at the National Bureau of Standards (now the National Institute of Standards and Technology), grants-in-aid were awarded to many universities for collecting new and improved data on compounds under study in their research projects and for the development of computer programs for analyzing diffraction data.

## 3. Structure and Operation of the JCPDS-ICDD

As indicated above, the JCPDS-ICDD consists of two parts: a permanent staff and voluntary scientific and technical advisers. This structure is somewhat parallel to ASTM, one of the parent organizations. The headquarters is now located in Newtown Square, PA (after a time of 20 years in Swarthmore, PA) where the 31 employees are responsible for producing and marketing the PDF. A bibliographer and several field-specific editors work under contract to locate and review the diffraction data which comprise the PDF. The volunteer members meet twice a year to discuss progress, to recommend new products and to consider ways to be of service to the diffraction community. The corporate business is conducted at the Annual Meeting in March of each year.

The office staff is composed of three major departments responsible for the PDF, the Editorial and Production Department, Research and Development Department, and the Sales and Marketing Department, plus the supporting Computer Department and Accounting Department. Although the printing of hard copy products is done outside, the mastering of the CD-ROM as well as most other operations is done in-house. As the computer became more important in the development of the PDF, more of the operations have been undertaken by the office staff. The General Manager is responsible for overseeing this operation and reports to the Board of Directors. The current General Manager is Ron Jenkins. Previous General Managers included A. S. Beward, R. G. Simard, A. W. Danko, J. W. Caum, J. Messick, and Dan Richardson.

The volunteers comprise over one hundred members of the JCPDS-ICDD. They are employed by industry, government and academia in about equal proportions. Also, 20 percent of these members are from outside the United States. The membership is structured into several special and two major operational committees and many subcommittees as listed in Table 2. The Technical Committee is responsible for recommending products, procedures and projects to the Board of Directors. The Finance Committee oversees the fiscal operations, proposes the annual budget, and recommends the disbursement of funds for projects. The Board of Directors, which is elected by the membership, is responsible for: setting the policies of the JCPDS-ICDD; final approval of all projects; approving the budget; and the monitoring the use of the funds. In addition to direct products involving the PDF, the JCPDS-ICDD sponsors a grant-in-aid program to assist researchers in the acquisition of new diffraction data and in educational courses for training new diffractionists. The current Chairman is R. L. Snyder. Previous Chairmen include W. P. Davey, H. W. Rinn, W. L. Fink, L. L. Wyman, J. D. Hanawalt, G. J. McCarthy, D. K. Smith, L. K. Frevel, and G. G. Johnson, Jr.

## 4. Collection and Evaluation of PDF Data

Since 1965, 2000 new patterns have been added yearly to the PDF. Each year about 1200 of these patterns are retrieved from the open literature, 300 are provided by regular supporters of the PDF, and 500 are provided by the grant-in-aid program sponsored by the JCPDS-ICDD. To collect the literature data, the JCPDS-ICDD employs a full-time bibliographer to scan through 120 journals on a regular basis and about 50 other journals on an intermittent basis. This search yields 2000 to 2500 patterns each year including some data which are obtained by encouraging authors to supply patterns which were implied in a paper but not published. Additional searches are contracted with computer-based abstracting services. These searches locate more than 2000 additional references and indicate other journals for the bibliographer to examine. The desired data are then extracted by the office staff and keyboarded for evaluation. The quality of these patterns is highly variable.

The patterns from the grants-in-aid projects are also keyboarded if they are not automatically supplied in computer-readable form. The compounds studied in these projects are coordinated with the needs of the PDF and the expertise of the grantee. All grant data are collected by experienced diffractionists under conditions which lead to accurate results, and the samples are well-characterized, including the crystal unit-cell and symmetry. These patterns are among the best that appear in the PDF, and their inclusion is one reason why the quality of the PDF improves with time. The compounds studied are usually part of a research project and represent materials of considerable current interest. In one year, the JCPDS-ICDD awarded over one-half million dollars in the grant-in aid program with the amount varying depending on available funds and suitable scientific requests.

After the data are obtained from the source, prior to their inclusion in the PDF, they are reviewed for appropriateness and quality. Historically, the evaluation was the responsibility of several editors who had considerable experience in powder diffraction and in the characterization of the materials involved. Early *d-I* data were replaced by newer data as the quality improved. The evaluation of quality, however, was subjective until 1965 when the *d*-values and intensities were first entered into computer-readable files. This step allowed the *d-I* data to be checked for consistency, and this checking has become more rigorous with time. The editors initially confirmed the indexing compatibility with the unit cell reported by the author, or attempted to determine an indexing solution which would explain all the observed *d*-spacings.

**Fig. 3:**
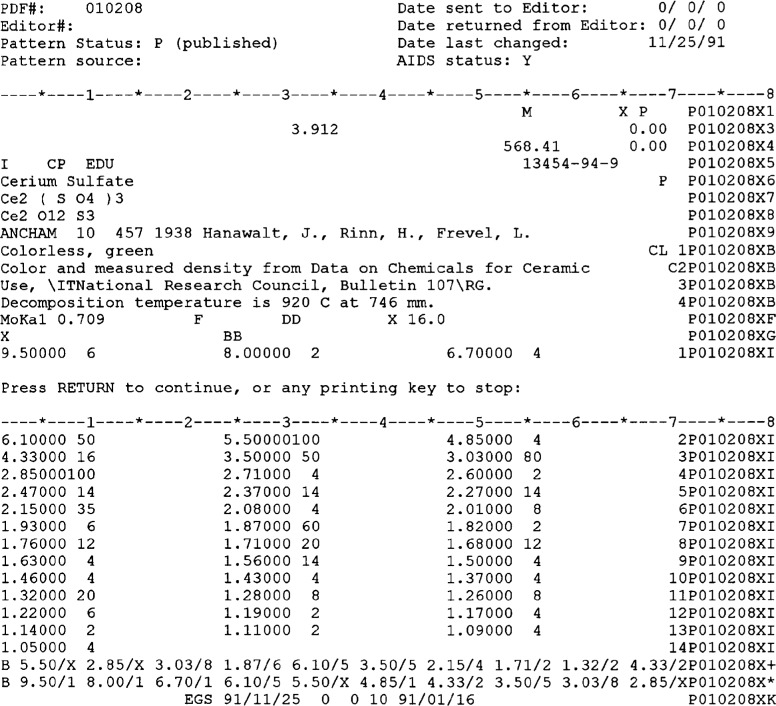
Display of the Ce_2_(SO_4_)_3_ data in the NBS*AIDS83 databse format from the 1989 PDF.

In early 1980, a sophisticated computer program for data entry and review was developed at the National Bureau of Standards (now the National Institute of Standards and Technology) under the name NBS*AIDS83, Mighell et al. (1983) [4]. This program was designed to evaluate data for both the Powder Diffraction File and for Crystal Data. A standardized procedure is followed which checks many aspects of the data as allowed by the information available. The *d*-spacings are evaluated by figures-of-merit (deWolff (1972) [5] and Smith and Snyder (1979) [6]) where unit cells are provided by the authors. These figures-of-merit are based on the average error in the observed diffraction angle compared to the calculated value from the refined unit cell and are used to assign the quality indicator for the data set. Crystallographic conditions are compared with the symmetry assignment provided by the author. The program also checks the consistency of the chemical formula by determining the formula weights and the density for comparison with measured values. The summary of this review is provided to the PDF editors to assist in their evaluation of the data. An example of NBS*AIDS83 information as stored is shown in Fig. 3.

The development of NBS*AIDS83 was the major step in initiating the comprehensive review of the older *d-I* data from Sets 1–32. All the data were re-evaluated, and the corrected data were entered into a master computer-readable database. The review also resulted in extensive changes to many individual, older *d-I* data where inconsistencies were detected. Because the NBS*AIDS83 program established a consistent set of rules for pattern evaluation, one result was the adjustment of many of the quality codes, generally downgrading many “*” patterns to “I” grade.

Currently all new data are entered into this master database following review and acceptance by the editors, maintaining it as the most current form of the PDF. Consequently, older hard copy forms of the PDF are out-of-date. This new database is now the source for all modern PDF products. All products, including the hard copy forms, are prepared directly from this master by appropriate computer formatting of the data. The computer-readable files allow the production of search manuals directly from the master file, minimizing the errors occurring with multiple data entry. Errors previously introduced by retyping are essentially eliminated. The book products are still based on the original “3×5” format used in the card format, but the image has been modified to allow more efficient use of the space. Changes such as the elimination of the three *d*-spacings across the top of the card image reflect the outdated use of these entries for a search system which was superseded by the first search manuals printed.

The editing of the data is the responsibility of the Editor-in-Chief, W. F. McClune, and previously three topical editors, B. Post, S. Weissmann, and H. F. Mc-Murdie, each of whom have been involved with the PDF for over 20 years. Other editors included W. P. Davey, G. W. Brindley, J. V. Smith, A. S. Beward, L. G. Berry, and M. E. Mrose. Prior to the implementation of the NBS*AIDS83 review, a copy of the original article containing the powder data was sent to the appropriate topical editor. This editor decided whether to continue with the review process and prepared a standard form with the extracted data. The office staff would then type the data on a master card and return a copy of the master to the editor for final review. The editor subjectively assigned one of the six quality marks, “*,” “I,” “ ”, “0,” “R,” or “C” to the data set on the basis of experience. As the approved patterns accumulated in the office, they were organized into units of 2000 for the yearly releases of the PDF.

With the introduction of the NBS*AIDS83 review, the editorial process underwent considerable revision. The potentially acceptable *d-I* data are now keyboarded into computer-readable form, and both the data and the NBS*AIDS83 output are supplied to the topical editor. The NBS*AIDS83 program provides the editor with sufficient information to make a final decision on the appropriateness of the new data to the PDF and to assign objectively the quality mark, which cannot be higher than the mark assigned by NBS*AIDS83. The present criteria for quality evaluation are given in Table 3. Once the topical editor has approved the pattern, the Editor-in-Chief checks for similar data in the PDF and assigns the data a position in the forthcoming PDF set. The acceptable data are then committed into the master database when the PDF set as the data are approved.

Some patterns receive additional review by task groups of the JCPDS-ICDD. Usually, this review occurs when a subfile product is being prepared, such as the Metals and Alloys Subfile. The minerals, though, are now reviewed on a yearly basis, so that when it is time to reissue the Minerals Subfile, the patterns are already in final form. This review consists of confirming the nomenclature for conformity with accepted usage and checking the presentation of the auxiliary information for completeness. For minerals, the nomenclature is governed by the Commission on New Minerals and Mineral Names of the International Mineralogical Association. Metals and alloys usually conform to the Pearson nomenclature system (Villars and Calvert (1987) [7]).

The importance of the NBS*AIDS83 review cannot be overemphasized. In addition to checking the items in Table 3, the program generates considerable derived information which is stored in the master database for future reference and products. The Crystal Data cell and determinative ratios, the reduced cell and its transformation matrix, derived crystal parameters, lists of the 10 lines with the strongest intensities and the 10 lines with the largest *d*-spacings, and the formula weight are added along with all the textual information on source, preparation, etc., keyboarded from the original manuscript. Fig. 3 shows an example of this information as recorded in the database. The original 2-*θ* data are preserved where provided by the author. All grant-in-aid data provide the experimental 2-*θ* values. Because of round-off problems, the 2-*θ* ’s obtained by back-calculating from reported *d*-values are not satisfactory for error analysis and the determination of the proper figures-of-merit.

The data which now appear in a PDF image are listed in Table 4. The primary data and all the other information available in the manuscript are included. Information from sources other than the principal references are added where appropriate. The goal of the PDF is to provide the user with as much information as possible to assist in the identification of the compound.

## 5. Current Products of the JCPDS-ICDD

The PDF is the principal product of the JCPDS-ICDD, but there are many supportive products. Table 5 is a comprehensive list of these products. Hard copy products are being replaced with computer-readable files to meet the needs of modern instrumentation. In 1986, the JCPDS-ICDD published the last set in card form. All future hard copy versions of the PDF will be in book form or as microfiche films. Computer-readable versions are available on a variety of media.

Search manuals, primarily the Alphabetical and Hanawalt manuals, will continue to be published in the foreseeable future. The Elemental and Interplanar Spacing Index (EISI), the latest search manual to be developed, is designed to assist the electron diffractionist by the use of chemistry and *d*-spacings but without the use of intensity values. It is a single-entry index which groups the compounds by largest *d*-spacing and chemistry and lists the ten lines with the largest *d*-spacings. The Fink manual, also designed primarily for electron diffraction, was last produced in 1982. Because it is a multiple-entry index, its size is too large to bind as one volume. Future editions will probably be coupled with computer-readable products or with subfiles where size is not a factor.

The computer-readable versions of the PDF are the most widely distributed form of the PDF today. At present there are two versions, the PDF-1 database with only PDF numbers, *d*-*I*’s and compound name, and the PDF-2 database with all the data present. The main media for distribution is the CD-ROM. The magnetic media are useful where the computer is programmed to search for matches in real time. Either the medium is searched directly or the PDF is restructured for faster implementation, and the magnetic media are amenable to this operation.

The CD-ROM is ideal for information storage and display on personal computers. The information packing density on a compact disk, is so high that the full PDF requires only about 70 % of the available space of a CD-ROM. The PDF already fills a magnetic tape at the highest density currently available. Maximum access time on the compact disk is about 1 s for a block of data and less than 0.1 s for a contiguous search. Thus, the CD-ROM is less suitable for real-time search/match operations. When the proper index files are prepared, it is an ideal medium for look-up and display of desired information. The JCPDS-ICDD has developed programs which accompany the CD-ROM product to access information in ways similar to what would be done if the information were in book form. The 1989 version of its PC program allowed searching on PDF number, chemistry, mineral names, and specific *d*-spacing combinations. More recent versions include the use of a Windows^1^ environment with additional searches such as a Hanawalt and Fink searches and matching of specific keys.

True real time search/match programs are not being developed by the JCPDS-ICDD because many are available from APD manufacturers or software developers. The JCPDS-ICDD considers itself a database producer, not a software house, but it does distribute programs developed by others. As soon as the PDF became available in computer-readable form in about 1963, several groups developed search programs. Some of these older programs are still being improved, and many new ones are being developed by equipment manufacturers and individual entrepreneurs. The JCPDS-ICDD will distribute any program in the public domain which will be supported by the authors. Presently, only three PDF programs fall into this category along with two for NIST Crystal Data and one for the Electron Diffraction Database. The JCPDS-ICDD maintains a public bulletin board and other Internet access, but does not, at the present allow searching of its files by users.

The JCPDS-ICDD also has developed materials for educating both new and experienced diffractionists. The Education Subcommittee has been one of the most active of its subcommittees. A regular schedule of workshop-type courses is presented in the United States, and additional courses are offered in conjunction with scientific meetings domestic and overseas. These programs have led to the development of workbooks which are also available for purchase at cost to encourage the in-corporation of powder diffraction methodology courses at colleges and universities. Working with the American Chemical Society, a short course was developed for audio instruction while following an illustrated manual. In addition to direct course-oriented material, the JCPDS-ICDD has assembled a Methods and Practices Manual which contains articles covering a wide variety of subjects from sample preparation to the evaluation of diffraction data. This manual is continually updated as new material becomes available. Most of the articles are reprinted from Powder Diffraction. Others are prepared to fill in specific subjects in the manual. This manual is available to purchasers of the PDF at cost and to others at an increased price.

In addition to the PDF, the JCPDS-ICDD has been the publisher of Crystal Data since 1963. This database of crystallographic unit-cell information is a useful supplement to the PDF in that it contains many more compounds than the PDF. In 1994, the NIST Crystal Data File had over 197 000 entries. This database is available both on magnetic tape and on CD-ROM. Cell matching search programs have been developed by Karen and Mighell (1987) [8] and Boolean programs by Harlow and Johnson [9] (personal communication, 1989), and are distributed by the JCPDS-ICDD. The NBS*AIDS83 program was developed to evaluate the data for the NIST Crystal Data File as well as for the PDF. The group responsible for maintaining the NIST Crystal Data File is under the auspices of the Office of Standard Reference Data Program at the National Institute of Standards and Technology.

Although most new releases of NIST Crystal Data will be in computer-readable form, there is a project in progress to bring all the mineral data up to date and print this information in a form similar to the books printed up to 1970. Because of the limited size of the mineral information, about 12 000 entries, this product is feasible. The program is being implemented by M. E. Mrose of the National Institute of Standards and Technology.

One other important product of the JCPDS-ICDD is the quarterly publication Powder Diffraction. This journal is devoted to disseminating throughout the world information of interest to the diffractionist. Articles range from theoretical to applied, and many new powder diffraction patterns are included. Appropriate articles are reprinted in the Methods and Practices Manual. In addition to the regular articles and data articles, the International Report section includes meeting reports, a computer section, and news of activities from the JCPDS-ICDD. The current editors are D. K. Smith, G. J. McCarthy, H. D. Hitchcock, B. C. O’Connor, H. Toraya, and J. W. Visser, with R. Jenkins as the Managing Editor.

## 6. The Computer-Readable PDF

The first computer-readable version of the PDF appeared in 1962 when the PDF numbers, *d*-*I*’s, and compound formulae were distributed on magnetic tape. That product is now known as PDF-1 (see Table 6). Computers of that time period were memory limited and, consequently, only essential data were included in this file. Magnetic tapes were the best way to present large blocks of data to computers. The availability of these data did encourage the development of search schemes for the identification of compounds. That stage marked the beginning of the computer age for the JCPDS-ICDD. Since that time, computer-based products have begun to take a more and more important position in diffraction analysis.

**Fig. 4:**
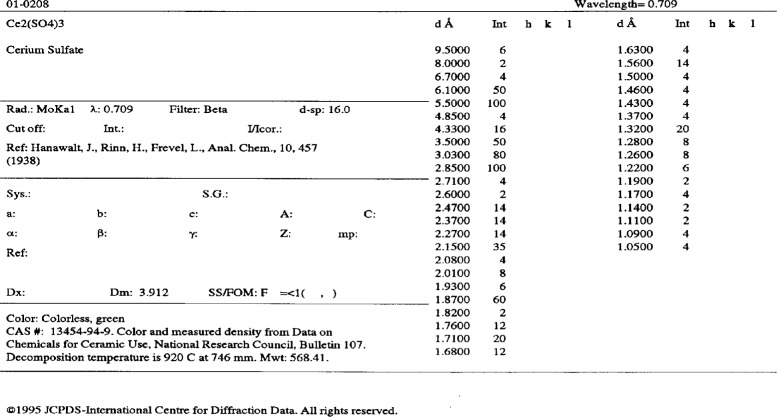
Display of the data image for Ce2(SO4)3 as printed from PCPDFWIN. Position data may be displayed in *d*, θ, 2-*θ*, Q or sin2-*θ* as desired.

It was not until about 1985 that the next major step was taken. PDF-2 was introduced as soon as all the historical data from the older sets of the PDF were keyboarded and checked. The contents of PDF-2 are listed in Table 7. Essentially all the information that appeared on a card image was now available in computer-readable form. Search programs still used only the information from PDF-1; PDF-2 was used to display the supporting information on the computer screen. The first version of PDF-2 was distributed on magnetic tape, but with the availability of the CD-ROM in the mid 1980s, it too was used as a medium for the PDF. Currently, the CD-ROM is one of the most popular media. Fig. 4 illustrates the card image as presented on a PC monitor.

## 7. Future Products

With the availability of computer-controlled diffractometers, it is now easy to collect and process digitized diffraction patterns. New experiments are now possible which analyze the peak shapes as well as position and intensity information. Such studies include structure analysis using profile fitting or whole-pattern fitting, crystallite size and strain, and quantitative analysis. It is evident that the future will need the digitized trace in addition to the current *d-I* data for complete diffraction studies. PDF-3 (a digitized full pattern database) is envisioned to meet these needs. A test product for clay minerals will be the first PDF-3 undertaking. The proposed contents of PDF-3 are described in Tables 8 and 9.

The many problems in the development of a new database such as PDF-3 are compounded because it is computer-based. The major problem is to define now what will be needed 10 or 20 years in the future. The central information in this database will be the digitized diffraction trace, but present-day instruments will evolve into the more sophisticated instruments of tomorrow and perhaps change the nature of the data that are needed. Current instruments introduce aberrations which may be minimized, and x-ray sources may become more nearly monochromatic in the future. Obviously, the most desired trace to preserve would be one with the source and instrument contributions deconvoluted to leave the sample component only. The state of the art is not quite ready for this step yet, so the alternative is to record both the instrument trace of the sample and the instrument trace of some standard taken under the same experimental conditions. The JCPDS-ICDD has instituted a project with their grant-in-aid recipients to preserve the digitized traces of the patterns they submit. The present standard is NBS SRM640 silicon, but a study of new possibilities has led to the recommendation of LaB6 as a good alternative to Si. A supply of LaB6 has recently been certified by the Office of Standard Reference Data of the National Institute of Science and Technology for profile applications. Now that this standard is available to diffractionists, the project of collecting digitized traces can begin in earnest.

Other problems remain in defining the angular range of the data to be collected and the step size to be used. As with the *d-I* data, many of the decisions will have to be made by the diffractionist based on the nature of the sample. The range of a pattern may vary considerably. Some compounds do not produce useful diffraction information beyond 90 ° 2-*θ* or even 60 ° 2-*θ* in some cases. Other compounds would allow useful information to be collected beyond the range of the diffractometer. Step size is more nebulous. For a reference pattern, the step size should be as small as the most demanding application requires. Step sizes between 0.01 ° and 0.05 ° 2-*θ* seem to be in common use. Most users prefer not to interpolate between data points, so the 0.01 ° 2-*θ* step for the reference patterns seems to be the acceptable choice.

Once the database of digitized traces becomes available, it will be necessary to transfer the traces from instrument to instrument. Thus a standardized file structure is necessary, and a universal file conversion program must be developed for each instrument. The JCPDS-ICDD is working on this difficulty and has tentatively decided to follow the IUCr in using the CIF/STAR format. CIF/STAR is available now for data archiving, and the PDF-3 will follow rules similar to those used in this format.

As the problems are solved, digitized traces will be accumulated by the ICDD, and when there is a significant number of these data, a set of PDF-3 patterns will be issued. The development of this database will be slow at first, but as the problems are overcome and the applications are extended, this database should become another product of the JCPDS-ICDD.

In addition to PDF-3, there are several other computer products that should appear within the next 2 years. The Metals and Alloys File is being extensively reviewed, and a product is now available. A complete Mineral File will probably appear about every 5 years.

The CD-ROM is currently the most popular computer media for obtaining the PDF. The NIST Crystal Data is already available on the CD-ROM and the Electron Diffraction Database can also be included on the CD-ROM.

## 8. Future of the JCPDS-ICDD

The renaissance of powder diffraction in the 1980s will continue into the next decade and probably beyond. New instrumentation will demand new products based on diffraction data. The JCPDS-ICDD is trying to anticipate these changes. The topic is always part of the agenda of the Technical Committee meetings. The Long-Range Planning Committee tries to look ahead 10 to 20 years to recommend new directions for endeavor.

New members for ICDD are continually being sought, and all members are encouraged to participate in as many activities as their time allows. Membership is limited only by interest. The meetings of the Technical Committee are open to anyone wishing to attend, and as new attendees indicate interest and participate in the activities of the JCPDS-ICDD, they are invited into membership. The JCPDS-ICDD financially supports three international meetings, EPDIC in Europe, Denver in the USA, and AXAA in Australia. Most JCPDS-ICDD meetings are held in the Philadelphia area because of the proximity to headquarters, but there are usually open ad-hoc sessions held at Congresses of the International Union of Crystallography and at other technical meetings from time to time. Information on the next scheduled meetings may be obtained at any time from JCPDS-ICDD headquarters.

The JCPDS-ICDD is responding to the change in its market from primarily domestic in the 1960s to primarily international in the 1990s. The market is now about equally divided among North America, Europe, and Asia with a small but growing influence from Australia. About 20 % of the members of the JCPDS-ICDD are from outside North America, and this number is increasing as more users become aware of the activities in which they can participate. The Board of Directors has overseas members, and there are consultants from all the technical areas of the world. Automation has certainly been one of the reasons for the rapid increase in the use of the PDF all over the world, but technological developments even in underdeveloped countries are having their effect. All diffractionists are invited to contact the JCPDS-ICDD to see how they can assist in the development of projects. Individuals need not attend the meetings to have a useful role in the future of the PDF and related products.

## Figures and Tables

**Table 1:** Distribution of patterns in the PDF Sets 1–45

The subfiles have also been updated with the following numbers of nondeleted patterns in each subfile:			
Common Phases	3197	Minerals	4091
Metals and Alloys	11 954	NBS (NIST)	1969
Forensic	3012	Zeolites	696
Education Package	1061	Superconductors	196
Cements	308	Explosives	149
Corrosive	13 635	Polymers	274
Pigment and Dyes	166	Pharmaceutical	17
The following total statistics are from Sets 1–45 (71 067 patterns):			
Inorganic	43 836	Organic	18 201
Dual	2584	Minerals	6446
Stars	7218	Calculated	3647
Indexed	16 135	Unknown quality	23 295
Poor quality	12 520	Deleted	8215

**Table 2:** Committees and Subcommittees of the JCPDS-ICDD

Board of Directors
Bylaws Committee
Crystal Data Management Board
Employee Benefits Committee
Ethics Committee
Grant-in-aid Committee
Journal Oversight Committee
Long-Range Planning Committee
Membership Committee
Member Travel Support Committee
Publication Review Committee
Scholarship Committee
Finance Committee
Budget Subcommittee
Sales and Marketing
Technical Committee
Ceramics Subcommittee
Crystal Data Subcommittee
Data Collection and Analysis Subcommittee
Diffraction Problems Subcommittee
Data Base Subcommittee
Education Subcommittee
Electron Diffraction Subcommittee
Minerals and Ceramics Subcommittee
Metals and Alloys Subcommittee
New Products Research and Development Subcommittee
Organic and Forensic Subcommittee
Pattern Calculation Subcommittee
PDF Editorial Staff
Search and Match Methods Subcommittee
Target Systems Subcommittee

**Table 3:** Several of the tasks performed by NBS*AIDS83

1. Formula checking including generation of an empirical formula.
2. Form of the chemical name and comparison with the formula for consistency.
3. Comparison of measured, calculated and estimated densities.
4. Validation of space group, aspect or lattice type with allowed symbols and assigned crystal system.
5. Generation of Pearson symbols for structure types.
6. Refinement of *d*-spacings and confirmation of indexing with extinction rules for space group.
7. Calculation of the Smith-Snyder and the deWolff figures-of-merit.
8. Assignment of the PDF quality mark using the initial assignment by the topical editor and the fit of the *d*-spacings to the refined unit cell.
9. Validation of all the editorial flags.
10. Errors and warnings are given when problems are detected.

**Table 4:** Documentation of PDF data

Primary
Experimental conditions
Sample description
Composition
Source
Derived *d*-I’s
Reference for data
Secondary
Crystallographic data
System
Unit cell
Space group
Density (calculated)
Internal standard
References for crystal information
Tertiary
Miller indices
Density (measured)
Figures-of-merit
Axial ratios
Crystal Data cell
Indices of refraction
Reference-intensity-ratio
References for physical property data
Quaternary
Synthesis or mineral locality
Stability
Structure type
Chemical analysis

**Table 5:** Products of the JCPDS-ICDD

The Powder Diffraction File—Inorganic and Organic
Card form (discontinued in 1987)
Microfiche form
Book form
Individual set
Historical data (re-edited)
NBS Associateship Pattern File
Mineral File
Metals and Alloys File
Common Phases File
Forensic File
Computer-readable
Magnetic tapes
PDF-1
PDF-2
Disks
CD-ROM
Search Manuals
Book form
Chemical Alphabetical
Hanawalt Method
Fink Method
Elemental and Interplanar Spacing Index
Software (provided without charge)
Johnson/Vand
Goehner/Garbauskas
Toby POWDER SUITE
Educational Materials
PDF Workbook
Mineral Workbook
Educational Package
Methods and Practices Manual
ACS Audio Short Course
Short Courses
Crystal Data
Book form
Identification File on magnetic tape
Minerals
CD-ROM
Software (provided without charge)
Karen/Mighell
Harlow/Johnson
Electron Diffraction Database
Magnetic tape
CD-ROM
Software (provided without charge)
Carr/Johnson
Journal
*Powder Diffraction*

**Table 6:** Contents of PDF-1

PDF #	*d*’s
Compound Name	*I*’s
Compound Formula	
Data Quality Mark	
Subfile Code	

**Table 7:** Contents of PDF-2

PDF #	*d*’s
Compound Name	*I*’s
Compound Formula	*hkl*’s
Data Collection Documentation	
Cell and Symmetry	
*Z* and Density	
Physical Properties	
Preparation and Purity	
Sources of Materials	
Figures-of-Merit	
*I*/*I*(cor)	
Data Quality Mark	
Subfile Code	

**Table 8:** Contents of PDF-3

PDF *#*	Digitized trace
Compound Name	*d*’s
Compound Formula	*I*’s
Data Collection Documentation	*hkl*’s
Digitized Trace Parameters	
Reference Sample Trace	
Cell and Symmetry	
*Z* and Density	
Physical Properties	
Preparation and Purity	
Sources of Materials	
Figures-of-Merit	
*I*/*I*(cor)	
Data Quality Mark	
Subfile Code	

**Table 9:** Digitized trace parameters

Start Angle	Finish Angle	Step Size
	I 2-*θ*	
	Reference Intensity Ratio	
	Instrument Parameters	
	Standard Reference Trace	

## Footnotes

1 Certain commercial equipment, instruments, or materials are identified in this paper to foster understanding. Such identification does not imply recommendation or endorsement by the National Institute of Standards and Technology, nor does it imply that the materials or equipment identified are necessarily the best available for the purpose.

## 9. Appendix A. Current Activities at the International Centre for Diffraction Data

Reported by Lawrence C. Andrews, formerly with the International Centre for Diffraction Data, 12 Campus Blvd., Newtown Square, PA 19073.

The Joint Committee on Powder Diffraction Standards (JCPDS, the founding organization of the International Centre for Diffraction Data) has supported and supplied the Powder Diffraction File (PDF) for more than 50 years. It is one of the oldest of currently active scientific databases.

### 9.1 Editorial Systems Upgrade

The editorial system to support production of the PDF has evolved over the years from the earliest system that worked by cut-and-paste with typewriters to punched card systems then to magnetic tape and finally to in-house production using a variety of computers. The data entry portion of this system is the latest to undergo substantial revision. In 1991, a program to replace some programmable Hewlett-Packard terminals was begun. The older system had been designed at NIST, and it was still functional. However, the system was difficult to modify, had only limited error checking, and the hardware was no longer fully supported by the manufacturer. It was decided to design a new system that used a PC, which would be much more easily modified and for which repairs could be done in-house. Paradox (a relational database package produced by Borland International) was chosen as the first system to be evaluated to see if it could produce an adequate system. Paradox was chosen because it fulfilled a number of important criteria, but it was the lowest-end product that seemed to have sufficient flexibility and enough features. It was assumed that it might not be possible to produce an adequate system in Paradox, but that if it was not possible, then lower-end systems would also fail. Another desirable feature of Paradox was that it was cheaper than the more powerful systems. For future expansions, Paradox also was supposed to have the ability to access databases maintained on other systems and across computer networks. These latter features have only recently been delivered by Borland, but we did not need them earlier. In fact, Paradox proved to be an excellent vehicle for producing the data entry system. It is sufficiently fast to run on 486 PCs; it has a good form generation system for generating screen forms for data entry to use; it has excellent facilities for checking input data for errors; it also has a powerful scripting language in which one can add more error checking that cannot be supported by the relatively simple methods provided by Paradox itself. The system has now been in operation for 4 years. Over time, a considerable number of improvements have been made in the system, so that it is both easier to use, and it also checks for whole classes of errors that could not be detected by either the older, terminal-based system or the first versions of the Paradox based system.

### 9.2 Investigations of Relational Database Systems

We early realized that the Paradox-based systems could be easily extended to hold an entire database, rather than the single pattern that is used in the data entry system. Although that led to a very non-normalized database, it proved to be an extremely useful system for the editors of the PDF. Currently, we are planning how to produce a more useful, normalized database. Although Paradox is being used currently, we are also looking at higher end systems. Paradox and other systems (such as Oracle, Sybase, SQL-Server, etc.) also are fully network capable in their current releases. This ability means that client/server systems are easily built using commercially available software. Data entry in just one of a number of database systems in use at ICDD. We have been reviewing how many other data sources are stored and used in an attempt to unify our handling of internal data.

### 9.3 A Tool for Converting Spacing Data

The Editorial Department has produced a tool for converting reliably between any two data types that we have found in the literature. Currently, 16 different data types are known to be in use (not counting the fact that data may be in any of several systems of units: Angstroms, nanometers, picometers, degrees, radians, rads, …) not to mention different levels of scaling. The entire tool works in extended precision so that any peculiar conversion does not lose accuracy. Considerable automated testing was possible by using the tool to do conversion from any randomly chosen data type to another randomly chosen data type with a randomly chosen input value. By using the output of the conversion as input to the same routine with the inverse conversion, it was possible to automatically check many of the features of the system. On input, each input data type is converted to the “standard” data type of its class (*d*-value, inverse *d*-value, or *θ*, 13 possible conversions, plus 3 unit conversions); in the middle, if the output data class differs from the input class, it is converted (only 6 possible conversions); in the output stage, the standard type is converted to the requested output type (13 possible conversion, plus 3 unit conversions). The tool also accumulates the derivatives of each of the subconversions and outputs the derivative of the output value with respect to the input value; this allows input uncertainties or standard deviations to be converted to the appropriate value for the output that was requested.

### 9.4 The Index Assignment Problem

The problem of assigning crystallographic indices to observed *d*-values when a unit cell is “known,” seems never to have been addressed in the technical literature. Although a number of powder diffraction programs must solve this problem, there is no known optimal solution.
